# General Public’s Perspectives on Medical Doctors and Local Clinics in South Korea

**DOI:** 10.3390/ijerph16112030

**Published:** 2019-06-07

**Authors:** Hyemin Jung, Min-Woo Jo, Hyun Joo Kim, Won Mo Jang, Sang Jun Eun, Jin Yong Lee

**Affiliations:** 1 Department of Health Policy and Management, Seoul National University College of Medicine, Seoul 03080, Korea; hyeminjung82@gmail.com; 2 Department of Preventive Medicine, University of Ulsan College of Medicine, Seoul 05505, Korea; mdjominwoo@gmail.com; 3 Department of Nursing Science, Shinsung University, Dangjin 31801, Korea; hyjkim2012@gmail.com; 4 Health Review and Assessment Committee, Health Insurance Review and Assessment Service, Wonju 26465, Korea; thomasjang2@gmail.com; 5 Department of Preventive Medicine, Chungnam National University College of Medicine, Daejeon 35015, Korea; 6 Department of Public Health and Community Medicine, Seoul Metropolitan Government—Seoul National University Boramae Medical Center, Seoul 07061, Korea

**Keywords:** local clinic, primary care, trust, satisfaction, South Korea

## Abstract

As patients in South Korea play the main role in choosing healthcare providers, understanding their attitudes and beliefs toward medical institutions is essential. This study evaluated the public’s perspectives on doctors and local clinics. A face-to-face interview survey was conducted with 1000 participants who represent the South Korean adult population. The questionnaire consisted of four domains: personal information; trust level for nine professionals, including doctors; healthcare utilization behavior and attitudes regarding local clinics; and assessment of local clinics. The trust level of the doctor was highest (3.16 out of 4) among nine professionals. 85.3% of the participants frequently visited local clinics because of accessibility. The main reason for visiting hospitals over local clinics was the belief that doctors employed at hospitals would be better qualified. People were generally satisfied with the service of local clinics but wanted more facilities and equipment. Among six attributes of primary care, “first contact” and “accessibility” got higher scores in importance and current performance. Lastly, the participants suggested that improving the quality of doctors was most important for the reinforcement of primary care. Efforts to consider public opinion should be made before establishing healthcare policies for primary care.

## 1. Introduction

In the traditional doctor–patient relationship, the doctor played a dominant role in treatment decision-making, while the patient took a more passive role. This was possible not only because the doctor monopolized medical information but also the major diseases were suitable for this “activity–passivity model”. However, the relationship has undergone drastic changes in the last two to three decades. As information asymmetry has been resolved with the development of the internet, more patients seek to participate in treatment decision-making. Furthermore, partnership with patients has been recently emphasized in chronic disease management, a major healthcare issue of modern society. As a result, the “mutual participation model” and “patient-centered medicine” are now required in various healthcare areas, especially in primary care [1]. In this context, establishing a sound doctor–patient relationship based on trust is one of the most important components of achieving better health outcomes. Many studies have reported that the trust in the doctor positively affected the patient’s compliance in primary care [2,3].

South Korean patients are guaranteed almost unlimited freedom of choice when selecting medical institutions. In South Korea, it is possible to meet a specialist in a hospital without a referral from a primary physician, regardless of the severity of the illness. As a result of the patients’ preference for the hospital, this freedom of choice has caused competition among local clinics, hospitals, and even public centers for outpatient service of primary care [4,5,6]. In this context, analyzing patients’ attitudes and beliefs about the primary care system should be a priority when establishing the role and position of primary care. Also, listening to the voice of stakeholders is of paramount importance for the successful implementation of any healthcare policy. There have been a few studies that discuss the views of general doctors, but few attempts have been made to identify the general public’s view regarding medical doctors or the primary care system of South Korea [7,8,9]. The purpose of this study was to assess the general public’s attitudes and beliefs about medical doctors and local clinics in South Korea as primary care providers.

## 2. Materials and Methods

A survey targeting the general population was conducted to collect their opinions about doctors and local clinics. Using the quota sampling method, 1000 adults (aged over 18) living in seven big cities in South Korea (Seoul (the capital city), Busan, Daegu, Incheon, Gwangju, Daejeon, and Ulsan) were recruited for this study. Quota sampling is regarded as an alternative to probability sampling when the expected response rate is low, and it is known that there is little difference in survey results [10,11,12]. We designated age, sex, and residence as quota controls based on the 2014 Population Census by Statistics Korea. The sampling error was ±3.1% at a 95% confidence level. Well-trained interviewers from a professional research agency (Gallup Korea) conducted face-to-face computer-assisted interviews with a structured questionnaire. The completed questionnaires were randomly selected and verified by supervisors. Compared with online surveys, face-to-face interview surveys provide the advantages of planned sampling from the population, low error rates in responses, and a relatively flexible questioning style [13,14]. In spite of temporal and cost restrictions, we adopted this methodology to assure representativeness and accuracy of responses.

The questionnaire designed for the general public consisted of four domains. The first domain collected personal information such as the participants’ sex, age, residency, education, self-reported standards of living, and accompanied chronic diseases. In the second domain, the participants were questioned on how much they trust medical doctors and other professionals. We converted the participants’ answers to a 4-point scale (really trust as 4, somewhat trust as 3, not really trust as 2, and never trust as 1) and calculated the mean score to compare the result with previous studies. Next, the general public’s behavior of healthcare utilization, their attitudes towards local clinics, and their levels of satisfaction were measured. Finally, the participants assessed the performance level of local clinics as primary care providers in the last domain. The overall and particular performance levels were measured with a 5-point Likert scale (Table 1). We calculated the frequency and the percentage for each answer using Excel 2013 (Microsoft Corporation, Redmond, WA, USA). This study was approved by the institutional review board of Seoul National University Boramae Medical Center (IRB No. 07-2016-04).

## 3. Results

### 3.1. General Characteristics of the Participants

The mean age of the participants was 43.6 years and exactly half of the participants were women. 43.9% of the participants lived in Seoul, the capital city of South Korea. The majority of participants (95.9%) had an education level of high school graduation or more, and reported their standards of living as middle (55.9%) or low (34.7%). 28.0% of the participants replied that they themselves or some of their family members had diagnosed chronic diseases (Table 2).

### 3.2. Trust Level of Various Professional Groups

When participants were asked how much they trust medical doctors and other professionals, 90.7% (really trust: 25.6%, somewhat trust: 65.1%) of the participants replied positively and only 9.3% (not really trust: 8.7%, never trust: 0.6%) said they distrust medical doctors. After converting their responses to a 4-point scale, medical doctor got the highest mean score (3.16), followed by nurse and teacher (both 3.08), Korean medicine doctor (3.07), pharmacist (3.03), dentist (3.01), accountant (2.70), and lawyer (2.62) (Table 3). Additionally, when the participants were asked to evaluate the image of the medical doctor, the majority agreed with statements such as “high level of professionalism” (91.5%), “dedicating themselves to their patients” (63.7%), “necessary for public health” (77.0%), “deserving respect as a profession” (72.6%), which implied they had a positive image of medical doctors as healthcare professionals. However, they evaluated the medical doctor negatively regarding illegality or corruption. Only 32.2% of them agreed with the statement “medical doctors are rarely related to illegality or corruption”.

### 3.3. Frequently Visited Medical Institute and the Reason for Visiting

Regarding the general public’s healthcare utilization behavior, 85.3% of participants answered that they frequently visited local clinics for basic healthcare. The main reason for visiting local clinics was the close proximity (71.0% of local clinic visitors). For the people who visited hospitals frequently for basic healthcare, the major reason for visiting was prior visitation (40.5% of lower-level hospital visitors) and trust in the quality of doctors (69.4% of higher-level hospital visitors) (Table 4).

### 3.4. General Public’s Experience and Thoughts regarding Local Clinics

To the question “Are you satisfied with the current service of local clinics?”, 75.8% (very satisfied: 5.0%, satisfied: 70.8%) replied that they were satisfied with current service, while only 2.8% (dissatisfied: 2.6%, very dissatisfied: 0.2%) were dissatisfied. The biggest reason for dissatisfaction was “lack of facilities and equipment” (51.7%), followed by “limited business hours” (50.2%), “long waiting times” (24.7%), “short counselling” (23.9%), “low quality” (16.3%), and “poor patient management system” (11.5%). Regarding the reason for choosing hospitals over local clinics, more than half of the participants referred to “better facilities and equipment” (58.2%). Other major reasons were “trust in the quality of doctors” (22.0%) and “well-organized care system” (17.7%). In addition, 74.8% of the participants believed the doctors in hospitals were more qualified than doctors in local clinics (Table 5).

### 3.5. General Public’s Assessment of Current Local Clinics as Primary Care Institutes

Finally, we asked the participants to select the most important role of local clinics and assess the adequacy of current local clinics as primary care institutes (Table 6). “First contact” (39.8%) and “Accessibility” (31.9%) were thought to be the most important roles of local clinics and both of them received higher performance scores (first contact: 3.63, accessibility: 4.03) compared with other roles (continuity: 3.58, coordination: 3.47, comprehensiveness: 3.31, family and community oriented care: 3.18). The overall performance score of local clinics was 3.62 out of 5.0. Finally, the participants responded that “improvements in the quality of doctors in local clinics” (37.3%) and “standardized clinical guidelines and care system” (35.1%) were principal to reinforcing local clinics performance as primary care institutes. 

## 4. Discussion

This study was conducted to analyze the general public’s view of medical doctors and local clinics as primary care providers. The first part of the survey showed that the general public trusted medical doctors more than other professionals and the absolute level of trust was quite high (3.16 out of 4). The image of doctors was generally positive except for one attribute related to illegality and corruption. Similarly, in a previous study, most of the patients replied that they were satisfied with their doctors’ clinical ability but dissatisfied with the doctors’ behavior of pursuing profit excessively [15]. It is well known that the relationship between doctor and patient, sometimes called “rapport”, has a significant influence on patients’ behaviors and attitudes [3,16,17]. Because the negative images of doctors could weaken the rapport and consequently result in poor compliance, various attempts to correct their negative images are needed.

The next questions were given to our participants to determine their healthcare utilization behavior and satisfaction. A large number of Korean people preferred hospitals to local clinics and the number of unnecessary hospital visits were up to 15% of the total outpatient visits [6]. In our study, 10.7% of the participants were revealed to be visiting hospitals for basic care. Although 85.3% visited local clinics for basic care, it was not because local clinics were believed to be suitable for basic care but because they were close in proximity. In the same context, patients who cared more about the quality of doctors seemed to visit hospitals. Interestingly, over two-thirds of the general public perceived that the quality of doctors in hospitals was better than that of doctors in local clinics, while most of the doctors perceived that there was no quality difference [9]. Although the Health Insurance Review and Assessment Service has been evaluating healthcare quality in South Korea since 2000, assessments of the quality of doctors in primary care are not yet available. More efforts to measure the quality of doctors are needed, including an evaluation program conducted by the providers themselves [18].

The overall satisfaction rate of local clinics among the general population was good. On the other hand, the general public was dissatisfied with the lack of facilities or equipment as well as the limited business hours of local clinics. South Korean local clinics were evaluated as having excessive facilities or equipment already according to OECD (Organization for Economic Co-operation and Development) health statistics [19]. The reason people felt there was a deficiency in facilities or equipment originates from a misunderstanding of the role of local clinics and primary care. Primary care is expected to take on the role of a gatekeeper in the healthcare delivery system, which promotes the rational use of medical resources and efficiency through suppressing unnecessary medical use [20]. Therefore, well-planned educational programs targeting the general public should be considered to correct their misunderstandings about primary care. 

The overall status of primary care in South Korea evaluated by the general public was better than that of medical doctors. The converted score of the general public was 72.4 (3.62 out of 5), while the mean score by medical doctors was 69.2 [9]. Among six key attributes of primary care, “first contact” and “accessibility” received relatively high scores in importance and current performance. This result was similar to that of medical doctors [9]. A qualitative study also indicated that both doctors and patients misunderstood the role of primary care, especially for “coordination”, “comprehensiveness”, and “continuity” [8]. There was also a large gap in opinion about the solution for strengthening primary care. The public pointed out that the quality of doctor, clinical guidelines, and management systems were the areas that needed to be improved most urgently. Only 2.5% of our respondents thought that reforming the reimbursement system for primary care was important, while most of the doctors emphasized it as a very critical point [9].

There were a few limitations to our study. First, as we included people who live in Seoul or six other major cities for convenience, the opinions of people who live in small cities or rural areas were excluded. So, there was a possible selection bias. Second, self-report bias or social desirability bias could occur because our interviewers always explained the purpose of the study prior to the interview. Despite these limitations, this study is valuable as it contains up-to-date population-based evidence about the general public’s view of doctors and primary care. Further studies objectively assessing the quality of primary care conducted by various providers will be needed in developing persuasive strategies.

## 5. Conclusions

When developing effective policies for the healthcare delivery system in South Korea, determining the opinion of patients is important because they have taken the initiative in deciding which level of medical institution to attend. According to our study, South Koreans were generally satisfied with local clinics, and their trust in medical doctors’ clinical ability was relatively high. However, they raised questions about the quality of the doctors working in the local clinics. Also, they were confused about the concept of primary care and the role of local clinics as primary care providers. Before making an effort to re-establish the healthcare delivery system in South Korea, people’s misperception of primary care and local clinics should first be corrected.

## Figures and Tables

**Table 1:** Summary of questionnaire.

Domains	Questions
Personal information	■ Age ■ Sex ■ Residence ■ Education ■ Self-reported standards of living ■ Whether having chronic diseases (self or family)
Trust level of professionals	■ The level of trust for each professional (medical doctor, Korean medicine doctor, dentist, pharmacist, nurse, lawyer, accountant, and teacher) ■ Image of medical doctors
Behavior, attitude, and satisfaction	■ Frequently visited medical institution ■ The reason for frequently visiting ■ The reason for choosing hospitals over local clinics ■ Awareness of gap between doctors in local clinics and hospitals ■ The level of satisfaction in local clinics ■ The reason for dissatisfaction in local clinics
Assessment of local clinics as primary care providers	■ Most important role of local clinics ■ Performance level of current local clinics (overall and for each attribute of primary care) ■ Reinforcement point to improve their performance

**Table 2:** General characteristics of the participants.

Variable	Category	*N*	(%)
Age	Mean ± standard deviation	43.6	±13.2
	19–29	190	(19.0)
	30–39	212	(21.2)
	40–49	232	(23.2)
	50–59	224	(22.4)
	≥60	142	(14.2)
Sex	Male	500	(50.0)
	Female	500	(50.0)
Residence	Seoul	439	(43.9)
	Busan	153	(15.3)
	Daegu	106	(10.6)
	Incheon	125	(12.5)
	Gwangju	62	(6.2)
	Daejeon	65	(6.5)
	Ulsan	50	(5.0)
Education	Below high school	41	(4.1)
	High school	450	(45.0)
	University or college	493	(49.3)
	Graduate school	16	(1.6)
Self-reported standards of living	High	84	(8.4)
	Middle	559	(55.9)
	Low	347	(34.7)
	Refuse to reply	10	(1.0)
Chronic disease	Self	76	(7.6)
	Family member	173	(17.3)
	Self and family member	31	(3.1)
	None	720	(72.0)

**Table 3:** Trust level of various professional groups.

Professional Group	Very Much (4)		Somewhat (3)		Not Really (2)		Never (1)		Score	
	*N*	(%)	*N*	(%)	*N*	(%)	*N*	(%)	Mean	±sd ^1^
Medical doctor	256	(25.6)	651	(65.1)	87	(8.7)	6	(0.6)	3.16	±0.59
Korean medicine doctor	201	(20.1)	671	(67.1)	120	(12.0)	8	(0.8)	3.07	±0.59
Dentist	183	(18.3)	654	(65.4)	150	(15.0)	13	(1.3)	3.01	±0.62
Pharmacist	155	(15.5)	720	(72.0)	121	(12.1)	4	(0.4)	3.03	±0.54
Nurse	180	(18.0)	722	(72.2)	93	(9.3)	5	(0.5)	3.08	±0.54
Lawyer	65	(6.5)	539	(53.9)	348	(34.8)	48	(4.8)	2.62	±0.68
Accountant	77	(7.7)	575	(57.5)	314	(31.4)	48	(4.8)	2.70	±0.66
Teacher	187	(18.7)	706	(70.6)	104	(10.4)	34	(3.4)	3.08	±0.55

^1^ sd: standard deviation.

**Table 4:** Frequently visited medical institute and the reason for visiting.

Questions	Local Clinics		H ^1^, General		H ^1^, Tertiary		Others		None	
	*N*	(%)	*N*	(%)	*N*	(%)	*N*	(%)	*N*	(%)
Frequently visited medical institute for basic care ^2^	853	(85.3)	74	(7.4)	36	(3.6)	27	(2.7)	10	(1.0)
Reason for frequently visiting ^3^										
Close to home	606	(71.0)	22	(29.7)	2	(5.6)	10	(37.0)	-	-
Prior visit	166	(19.5)	30	(40.5)	7	(19.4)	9	(33.3)	-	-
Trust in the quality of doctors	52	(6.1)	16	(21.6)	25	(69.4)	4	(14.8)	-	-
Kindness	26	(3.0)	6	(8.1)	1	(2.8)	3	(11.1)	-	-
Others	2	(0.2)	0	(0.0)	1	(2.8)	1	(3.7)	-	-
Do not know	1	(0.1)	0	(0.0)	0	(0.0)	0	(0.0)	-	-

^1^ H: Hospitals. ^2^ Denominator of percentage in this row is the total participants (*N* = 1000). ^3^ Denominator of percentage in each column is the sum of cells.

**Table 5:** General public’s experience and thoughts regarding local clinics.

Questions	Answers	
Are you satisfied with the current service of local clinics?	*N*	(%)
Very satisfied	50	(5.0)
Satisfied	708	(70.8)
So so	214	(21.4)
Dissatisfied	26	(2.6)
Very dissatisfied	2	(0.2)
What is the reason for your dissatisfaction? ^1^	*N*	(%)
Lack of facilities and equipment	517	(51.7)
Limited business hours	502	(50.2)
Long waiting times	247	(24.7)
Short counselling	239	(23.9)
Low quality	163	(16.3)
Poor patient management system	115	(11.5)
Too far from home	88	(8.8)
Unkind staff	59	(5.9)
Too expensive	18	(1.8)
Other	6	(0.6)
None	8	(0.8)
What would be the main reason for choosing hospitals over local clinics?	*N*	(%)
Better facilities and equipment	582	(58.2)
Trust in the quality of doctor	220	(22.0)
Well-organized care system	177	(17.7)
Advertisement via mass media	13	(1.3)
Responsibility when medical conflict occurred	8	(0.8)
Do you think there is a quality gap between doctors in local clinics and hospitals?	*N*	(%)
The quality of doctors in local clinics might be better	68	(6.8)
The quality of doctors in hospitals might be better	748	(74.8)
There might be little difference	184	(18.4)

^1^ Multiple answers were allowed for this question.

**Table 6:** General public’s assessment of current local clinics as primary care institutes.

Questions	Answers	
What do you think is the most important role of local clinics?	*N*	(%)
First contact	398	(39.8)
Accessibility	319	(31.9)
Continuity	150	(15.0)
Coordination	104	(10.4)
Comprehensiveness	28	(2.8)
Family and community oriented	2	(0.2)
Do not know	1	(0.1)
Please rate the performance level of current local clinics for each attribute.	Mean	±sd ^1^
Overall	3.62	±0.57
First contact	3.63	±0.70
Accessibility	4.03	±0.73
Continuity	3.58	±0.76
Coordination	3.47	±0.74
Comprehensiveness	3.31	±0.80
Family and community-oriented care	3.18	±0.79
What should be reinforced to improve local clinics’ performance?	*N*	(%)
Improvement in the quality of doctors	373	(37.3)
Standardized clinical guidelines and care system	351	(35.1)
Efficient healthcare delivery system	164	(16.4)
Straightening out public’s misconception about local clinic	58	(5.8)
Constriction of outpatient care in hospitals	25	(2.5)
Reforming the reimbursement system for local clinics	25	(2.5)
Others	3	(0.3)
None	1	(0.1)

^1^ sd: standard deviation.
